# The role of cognitive and social leisure activities in dementia risk: assessing longitudinal associations of modifiable and on-modifiable risk factors

**DOI:** 10.1017/S204579602100069X

**Published:** 2022-01-11

**Authors:** L. A. Duffner, K. Deckers, D. Cadar, A. Steptoe, M. de Vugt, S. Köhler

**Affiliations:** 1 Alzheimer Centrum Limburg, School for Mental Health and Neuroscience (MHeNs), Department of Psychiatry and Neuropsychology, Faculty of Health, Medicine and Life Sciences, Maastricht University, The Netherlands; 2 Department of Behavioural Science and Health, Institute of Epidemiology and Health, University College London, London, UK

**Keywords:** ageing, dementia, cohort study, epidemiology, public health, risk factors, leisure time, lifestyle

## Abstract

**Aims:**

With the projected surge in global dementia cases and no curative treatment available, research is increasingly focusing on lifestyle factors as preventive measures. Social and cognitive leisure activities are promising targets, but it is unclear which types of activities are more beneficial. This study investigated the individual and joint contribution of cognitive and social leisure activities to dementia risk and whether they modify the risks associated with other potentially modifiable and non-modifiable risk factors.

**Methods:**

We used data from the English Longitudinal Study of Ageing (ELSA) from 7917 participants, followed up from 2008/2009 (Wave 4) until 2018/2019 (Wave 9) for incident dementia. Self-reported baseline cognitive activities (e.g. ‘reading the newspaper’), the number of social memberships (e.g. being a member of a social club) and social participation (e.g. ‘going to the cinema’) were clustered into high and low based on a median split. Subsequently, their individual and joint contribution to dementia risk, as well as their interaction with other dementia risk factors, were assessed with Cox regression models, adjusting for age, sex, level of education, wealth and a composite score of 11 lifestyle-related dementia risk factors.

**Results:**

After a median follow-up period of 9.8 years, the dementia incidence rate was 54.5 cases per 10.000 person-years (95% CI 49.0–60.8). Adjusting for demographic and other lifestyle-related risk factors, higher engagement in cognitive activities (HR = 0.58; 95% CI 0.40–0.84), a greater number of social memberships (HR = 0.65; 95% CI 0.51–0.84) and more social participation (HR = 0.71; 95% CI 0.54–0.95) were associated with lower dementia risk. In a joint model, only engagement in cognitive activities (HR = 0.60; 95% CI 0.40–0.91) and social memberships (HR = 0.75; 95% CI 0.56–0.99) independently explained dementia risk. We did not find any interaction with other modifiable and non-modifiable risk factors.

**Conclusions:**

Engagement in cognitive and social leisure activities may be beneficial for overall dementia risk, independent of each other and other risk factors. Both types of activities may be potential targets for dementia prevention measures and health advice initiatives.

## Introduction

Dementia is a public health priority due to rising numbers in a continuously ageing world population (World Health Organization, 2018). Next to the emotional and care burden placed upon people with dementia and their families, dementia is also associated with major costs for society (Patterson, 2018). While certain risk factors such as age, sex, genetic predisposition and low parental socioeconomic status (SES) are non-modifiable, overall dementia risk is not set in stone (Livingston *et al*., 2020; Steyaert *et al*., 2020). According to the 2020 report of the Lancet Commission on Dementia Prevention, Intervention and Care, 40% of the risk is changeable by means of tackling modifiable risk factors (Livingston *et al*., 2020). These, amongst others, include lifestyle factors such as adherence to a healthy (Mediterranean) diet, physical activity, as well as the timely management of depression, hypertension, hypercholesterolemia and diabetes (Petersson and Philippou, 2016; Wium-Andersen *et al*., 2019; Thomassen *et al*., 2020; Litke *et al*., 2021).

A growing body of research also points to the engagement in cognitively stimulating leisure activities as protective against cognitive decline and dementia. For instance, a systematic review found that engagement in cognitively stimulating leisure activities in middle adulthood as well as in late life was associated with a lower risk for developing Alzheimer's disease and other dementias (Stern and Munn, 2010). Research also supports evidence for the relationship between social activities and dementia risk. A meta-analysis showed that less frequent social contacts, higher levels of loneliness and lower levels of social interaction were related to higher dementia incidence (Kuiper *et al*., 2015).

While mentally stimulating and social leisure activities appear to be promising factors for curbing cognitive decline and decreasing dementia risk, some studies with long follow-up suggest that reversed causality might be at play, where impending dementia leads to disengagement from cognitive and social activities (Foubert-Samier *et al*., 2014; Floud *et al*., 2021). Furthermore, activities are often correlated, with cognitive activities having a substantive social component and *vice versa*. As both types of activities are usually studied in isolation, independent effects have thus far not been well established. In addition, only a few studies investigated their potential to moderate, or even compensate for, the influence of other modifiable and non-modifiable (age, sex, education, socioeconomic position) risk factors. A recent study assessed whether sex and marital status modified the association between engagement in leisure activities and dementia risk (Almeida-Meza *et al*., 2021). Through independent analyses for various activities, the authors found that for specific subgroups of participants, some activities (reading the newspaper for females and mobile phone usage for males) independently predicted dementia risk.

Despite the fact that many studies included additional risk factors (including lifestyle) as covariates, they did not separately assess whether their associations could be moderated by the engagement in cognitive and social activities (Fancourt *et al*., 2018, 2020; Almeida-Meza *et al*., 2021). However, in order to be able to tailor dementia prevention programmes to especially at-risk people, the potential of activity engagement for modifying the association between lifestyle risk and dementia has to be examined in more depth. While one study found that stimulating activities, as measured by a composite of education, occupation and leisure activities, may buffer the impact of diabetes on dementia risk (Marseglia *et al*., 2020), there are, to date, no studies including a broader set of lifestyle factors.

The current study, therefore, aims at (a) investigating the independent as well as the joint contribution of cognitive and social leisure activities to dementia risk and (b) to study their potential to moderate the role of other modifiable and non-modifiable risk factors of dementia. We expect that higher numbers of cognitive and social leisure activities have independent associations with dementia and that they may moderate the influence of several other risk factors such as low education, low wealth and lifestyle factors.

## Methods

### Study design and participants

Data were extracted from the English Longitudinal Study of Ageing (ELSA), a multi-centre panel study initiated in 1998 (Phelps *et al*., 2020), and representative of the general English population aged 50 and older. ELSA data collection takes place in 2-year intervals, and amongst others, entails information about health and social well-being, lifestyle, psychological factors and economic status. Details about the sampling and data collection procedures have already been specified elsewhere (Steptoe *et al*., 2013). This study used data of Wave 4 (2008/2009; *n* = 11 050) as the baseline, as this wave is the most complete with regard to information about lifestyle factors. In case of unavailability of variables of interest, data of Wave 3 (2006/2007) or Wave 5 (2010/2011) were used alternatively. The final assessment took place at Wave 9 (2018/2019), yielding a maximum observational period of 11 years. Prevalent dementia cases at Wave 4 (*n* = 213) and people who ceased their participation after Wave 4 (*n* = 890) were excluded from further analysis. Furthermore, those with information on fewer than 11 factors of the ‘LIfestyle for BRAin health’ (LIBRA)-score (Deckers *et al*., 2015) (*n* = 2006) and missing information about education (*n* = 8) and invalid sampling weights (*n* = 16) were excluded. The final analytical sample included 7917 participants. Information about panel attrition between Wave 4 and Wave 9 can be found in online Supplementary Table 1.

### Instruments and measures

#### Dementia ascertainment

Dementia diagnoses were ascertained through either physician diagnosis of dementia or Alzheimer's disease (self or informant-reported) or the total score on the 16-item Informant Score Questionnaire on Cognitive Decline in the Elderly (IQCODE; Jorm, 1994). The IQCODE is an informant-based dementia screening tool, in which a family member or caregiver is asked to rate changes in cognitive functioning of a person over a 2-year period (e.g. ‘*Ability to remember the own address or telephone number as compared to the previous interview*’). The IQCODE has shown satisfactory psychometric properties across various populations (see Jorm, 2004 for a review). Possible scores range from 1 (*much improved*) to 5 (*much worse*). The chosen cut-off of an average score of 3.38 is regarded as suggestive for pathological cognitive decline and has proven an appropriate trade-off between sensitivity (0.82) and specificity (0.84; Quinn *et al*., 2014).

#### Cognitive and social activity engagement

Information about engagement in cognitive activities was collected by questionnaires administered during the Wave 4 assessment. Questions about activities were asked in a closed manner. Cognitive activities included reading the newspaper, having a hobby, having taken a holiday or owning a mobile phone. Social activities were assessed in two ways. On the one hand, the membership of various clubs or societies, such as a political party, tenant groups, religious groups or charitable associations (social memberships), was assessed. On the other hand, social participation was inferred by questions about how frequently participants visited an art gallery, museum, theatre, concert or cinema, and how often the respondent ate out. Choices ranged from ‘*twice a month*’ to ‘*never*’. The number or frequency of engagement of the respective activities was summed up to obtain total scores for cognitive activities (theoretical range 0–7), social memberships (theoretical range 0–8) and social participation levels (theoretical range 0–20), respectively. For the present study, scores were clustered into high and low, based on a median split. Social memberships and social participation were used as separate variables for the further course of the analysis.

#### Other lifestyle factors

Additional modifiable lifestyle factors were assessed using the LIBRA score (Deckers *et al*., 2015) based on data from Wave 4. The LIBRA score is a well-validated summary score, based on the relative contributions of 12 risk and protective factors for dementia: physical inactivity, smoking, alcohol use, diet, hypertension, cognitive activity, depression, obesity, diabetes, coronary heart disease, kidney disease (not available/measured in ELSA) and hypercholesterolemia (Vos *et al*., 2017; Deckers *et al*., 2018, 2019*a*, 2019*b*, 2020; Schiepers *et al*., 2018). The complete LIBRA index ranges from −5.9 to +12.7, with higher scores representing higher dementia risk. For the purpose of this study, however, the measure of cognitive activity was excluded from the weighting, yielding an adapted theoretical range of −2.7 to +12.7 for the adjusted LIBRA (LIBRA_adj_). A detailed summary of the construction of the LIBRA_adj_ scores can be found in online Supplementary Table 2 (adapted from Deckers *et al*., 2019*a*).

#### Socioeconomic status

The total net wealth of a household was considered as a proxy for resource-based SES at Wave 4. This was derived by summing up the value of possessions and assets and subtracting open mortgages and payments. The resulting relative amounts were then divided into tertiles, representing low, medium and high wealth.

#### Demographics

Information on age, sex and level of education was collected through questionnaires administered at Wave 4. The level of education was based on the highest academic degree obtained. Educational attainments were then categorised into *low* (no formal education), *medium* (ordinary level or secondary education) and *high* (college/university) education.

#### Ethical approval

ELSA received ethical approval from the National Health Service Multicentre Research and Ethics Committee and the University College London Research Ethics Committee. All participants provided written informed consent.

#### Statistical analysis

The difference in demographic information, cognitive activities, social memberships, social participation, wealth, education and LIBRA_adj_ scores between people with and without dementia was assessed by independent samples *t*-tests or *χ*^2^-tests. In case of violation of assumptions, a non-parametric alternative (Mann–Whitney *U* test) was chosen. For subsequent analyses, only cases without missing information on the specific activity variable were considered. Cox proportional hazards regression was used to study the individual contribution of cognitive activities, social memberships and social participation on dementia risk, controlling for age, sex, LIBRA_adj_ score, net-wealth and education, resulting in hazard ratios (HRs) and their 95% confidence intervals (CIs). In the separate analyses per activity type, variables were added in a stepwise manner, starting with a minimally (age and sex) adjusted model (model 1), followed by a model that was additionally adjusted for education (model 2). A third model then further adjusted for wealth (model 3), followed by the full model also adjusting for LIBRA_adj_ scores (model 4). Covariates were added in this specific order to assess changes to the association between the specific activity variable and dementia by the particular modifiable and non-modifiable risk factor. Model 4 is the main model used for testing the hypotheses of interest.

We then assessed the interactions between the three activity domains following the same approach as described above (models 1–4). Finally, the separate interactions between cognitive activities, social memberships or social participation with other modifiable (LIBRA_adj_) and non-modifiable (sex, education and net-wealth) risk factors were subsequently examined in the same manner. In all analyses, dementia was treated as the failure event. Survival time was defined as the period from birth until either the onset of dementia, the last interview or death (whichever came first). By defining survival time this way, age was considered in the time scale for all analyses. We used the Schoenfeld Residuals Test (Schoenfeld, 1982) and clog-log plots to examine the proportional hazard assumption. Furthermore, a sampling weight (baseline cross-sectional weight) was used in order to back-weight estimates from the analytical sample to the total sample to minimise selection bias. In ELSA, participants can also be selected from the same household, and we therefore used a robust sandwich estimator to adjust standard errors for household clusters. All analyses were conducted in Stata (version 14.2; StataCorp, 2015), and the level of statistical significance was *p* < 0.05 in two-sided tests.

## Results

### Sample characteristics

Participants were followed up for a median duration of 9.8 years (IQR = 4.1 years). Until the end of the observation period, 360 individuals (4.5%) had developed dementia, yielding an incidence rate of 54.5 cases per 10 000 person-years (95% CI 49.0–60.8). The mean age was 65.9 years (s.d. = 9.5 years; range: 50–99), and 3349 participants (45.1%) were female. Baseline characteristics are summarised by dementia status in Table 1.

**Table 1.** Baseline characteristics of ELSA Wave 4 participants by incident dementia status

	Overall	No dementia	Dementia	*p*-Value
*N*	7917	7557	360	
Age, mean (s.d.)	65.9 (9.5)	65.5 (9.3)	75.4 (9.1)	**<0.001**^1^
Female, *n* (%)	4349 (54.9%)	4128 (54.6%)	221 (61.2%)	**0.014**^1^
*Level of education, n (%)*				
Low	3230 (40.8%)	3031 (40.1%)	199 (55.3%)	
Medium	2128 (26.9%)	2045 (27.1%)	83 (23.1%)	**<0.001**^1^
High	2559 (32.3%)	2481 (32.8%)	78 (21.7%)	
*Net-wealth, n (%)*				
Low	2511 (31.7%)	2362 (31.3%)	149 (41.4%)	
Medium	2714 (34.3%)	2593 (34.3%)	121 (33.6%)	**<0.001**^1^
High	2692 (34.0%)	2602 (34.4%)	90 (25.0%)	
Level of cognitive activity engagement, *n* (%)				
Low	4977 (68.3%)	4690 (67.4%)	287 (87.5%)	
High	2314 (31.7%)	2273 (32.6%)	41 (12.5%)	**<0.001**^1^
Level of social memberships, *n* (%)				
Low	4077 (58.0%)	3875 (57.7%)	202 (66.2%)	
High	2950 (42.0%)	2847 (42.3%)	103 (33.8%)	**0.003**^1^
Level of social participation, *n* (%)				
Low	3301 (48.9%)	3111 (48.1%)	190 (67.9%)	
High	3451 (51.1%)	3361 (51.9%)	90 (32.1%)	**<0.001**^1^
LIBRA_adj_ score, mean (s.d.)^2^	1.5 (2.4)	1.4 (2.4)	2.2 (2.4)	**<0.001**^1^

s.d., standard deviation; LIBRA_adj_ score, LIfestyle for BRAin health score without the weight for cognitive activity.

1 Statistically significant at *p*≤0.05

2 Observed range −2.7 to 10.3 (theoretical range: −2.7 to 12.7).

Questionnaires about leisure activity engagement have been returned by approximately 80% of the total sample. There were 644 participants with missing information on cognitive activities. They more likely had lower LIBRA_adj_ scores (*z* *=* 6.45; *p* < 0.001), and lower net-wealth (*z* *=* 7.40; *p* < 0.001) in comparison to people without missing information. Furthermore, 908 participants had missing information on social memberships. They were more likely to have dementia (*χ*^2^ = 7.87; *p* = 0.005), were younger (*z* *=* −2.61; *p* = 0.01), had a lower education (*χ*^2^ = 57.39; *p* < 0.001) and lower net-wealth (*t* = 7.02; *p* < 0.001) than those without missing information. People with missing information on social participation (*n* = 1183) more likely had dementia (*χ*^2^ = 19.28; *p* < 0.001), were older (*z* *=* −8.62; *p* < 0.001), were more often female (*χ*^2^ = 3.90; *p* = 0.048), had a lower education (*χ*^2^ = 82.17; *p* < 0.001), had higher LIBRA_adj_ scores (*z* = −2.49; *p* = 0.013) and a lower net-wealth (*t* *=* 6.46; *p* < 0.001).

### Individual associations of cognitive activities, social memberships and social participation with dementia risk

**Fig. 1.**
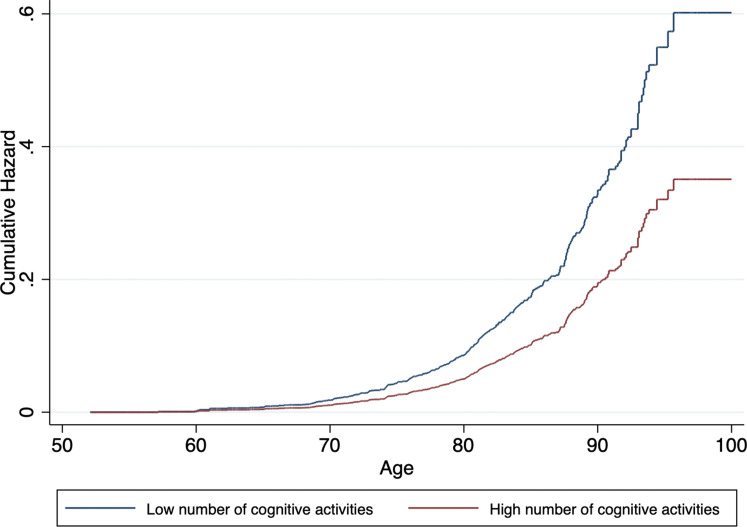
Cumulative hazard estimates of incident dementia by baseline level of cognitive activities (low *v*. high) adjusted for age, sex, level of education, wealth and LIBRA_adj_ scores.

In model 1, participation in a higher number of cognitive activities was associated with lower risk for dementia. This association remained significant in model 4. Table 2 presents a more detailed description of the stepwise analysis. Cumulative hazard curves by level of cognitive activities, adjusted for all covariates, can be found in Fig. 1.

**Table 2.** Stepwise Cox proportional hazard regression for the individual contribution of cognitive activity, social memberships and social participation

Model	Variable	HR (s.e.)	95% CI	*p*-Value
Model 1	Low number of cognitive activities	[Reference]		
	High number of cognitive activities	0.47 (0.08)	0.33–0.67	**<0.001**^1^
Model 2	Low number of cognitive activities	[Reference]		
	High number of cognitive activities	0.49 (0.09)	0.35–0.70	**<0.001**^1^
Model 3	Low number of cognitive activities	[Reference]		
	High number of cognitive activities	0.54 (0.10)	0.37–0.78	**0.001**^1^
Model 4	Low number of cognitive activities	[Reference]		
	High number of cognitive activities	0.58 (0.11)	0.40–0.84	**0.004**^1^
Model 1	Low number of social memberships	[Reference]		
	High number of social memberships	0.56 (0.07)	0.44–0.72	**<0.001**^1^
Model 2	Low number of social memberships	[Reference]		
	High number of social memberships	0.57 (0.07)	0.45–0.73	**<0.001**^1^
Model 3	Low number of social memberships	[Reference]		
	High number of social memberships	0.61 (0.08)	0.47–0.78	**<0.001**^1^
Model 4	Low number of social memberships	[Reference]		
	High number of social memberships	0.65 (0.09)	0.50–0.84	**0.001**^1^
Model 1	Low frequency of social participation	[Reference]		
	High frequency of social participation	0.59 (0.08)	0.45–0.78	**<0.001**^1^
Model 2	Low frequency of social participation	[Reference]		
	High frequency of social participation	0.61 (0.09)	0.46–0.81	**0.001**^1^
Model 3	Low frequency of social participation	[Reference]		
	High frequency of social participation	0.65 (0.09)	0.49–0.86	**0.003**^1^
Model 4	Low frequency of social participation	[Reference]		
	High frequency of social participation	0.71 (0.10)	0.54–0.95	**0.021**^1^

HR, hazard ratio; s.e., standard error; LIBRA_adj_, LIfestyle for BRAin health score without the weight for cognitive activity.

Model 1 = age and sex; Model 2 = model 1 + education; Model 3 = model 2 + wealth; Model 4 = model 3 + LIBRA_adj_.

1 Statistically significant at *p*≤0.05.

**Fig. 2.**
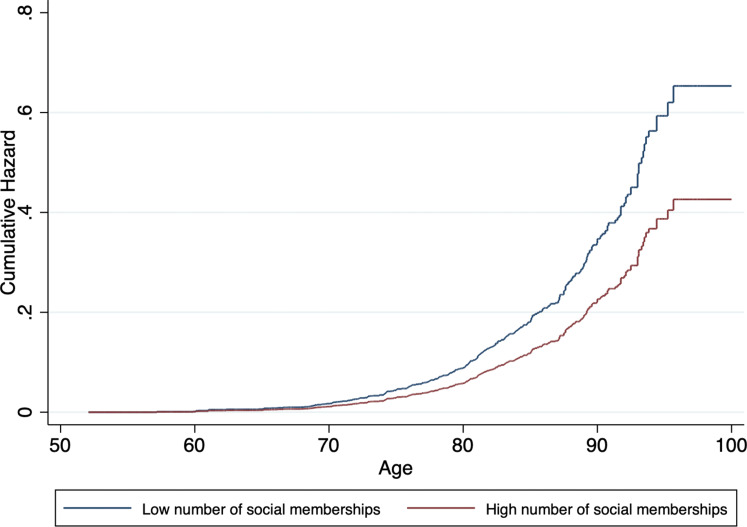
Cumulative hazard estimates of incident dementia by baseline level of social memberships (low *v*. high), adjusted for age, sex, level of education, wealth and LIBRA_adj_ scores.

**Fig. 3.**
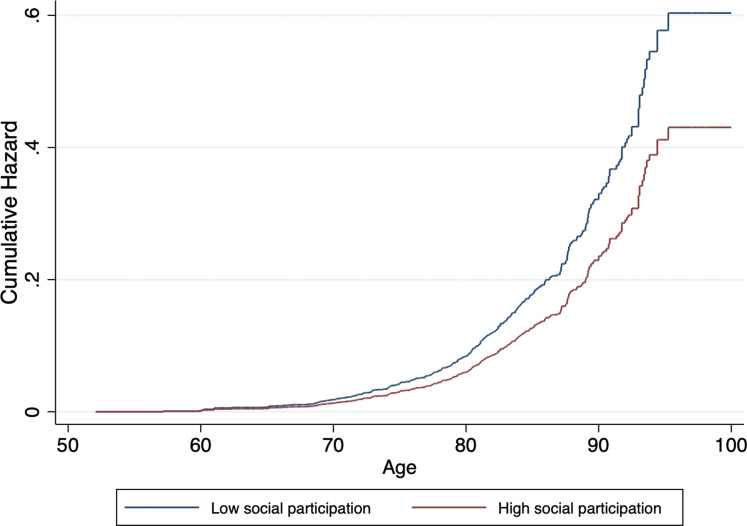
Cumulative hazard estimates of incident dementia by baseline level of social participation (low *v*. high), adjusted for age, sex, level of education, wealth and LIBRA_adj_ scores.

A higher number of social memberships was significantly associated with lower dementia incidence in model 1. This association was still observed in model 4. Similarly, more social participation was associated with lower dementia risk across all models. Cumulative hazard curves for social memberships and social participation, adjusted for covariates, can be found in Figs 2 and 3, respectively. In general, associations became attenuated but remained significant with incremental adjustment for non-modifiable and modifiable risk factors. In particular, wealth and LIBRA_adj_ scores seemed to partly account for the associations, with the most substantial reduction in effect size for the relation between social participation and incident dementia.

### The unique contribution of cognitive activities, social memberships and social participation

Table 3 presents multiple Cox proportional hazard models fitted to assess the joint contribution of the activity domains on dementia risk. The full model (model 4) thus included the three activities as predictors and age, sex, education, wealth and LIBRA_adj_ scores as covariates. As compared with those reporting low engagement in cognitive activities, those high in cognitive activity showed a 40% lower risk for dementia (HR = 0.60; 95% CI 0.40–0.91; *p* = 0.017). Similarly, social memberships were still negatively associated with dementia risk though the association became modestly attenuated (HR = 0.74; 95% CI 0.56–0.99; *p* = 0.039), corresponding to a 26% lower risk in the high as compared to the low social membership group. Social participation, on the other hand, did not significantly predict dementia risk (HR = 0.81; 95% CI 0.60–1.10; *p* = 0.172).

**Table 3.** Interaction between levels of cognitive activity, social memberships, social participation and other dementia risk factors

Interaction	Level	HR (s.e.)	95% CI	*p*-Value
Cognitive activities × sex^1^	High number of cognitive activities × female	0.97 (0.33)	0.49–1.90	0.917
Cognitive activities × education^2^	High number of cognitive activities × medium	2.04 (0.93)	0.84–4.99	0.117
	High number of cognitive activities × high	0.84 (0.41)	0.33–2.16	0.719
Cognitive activities × wealth^3^	High number of cognitive activities × medium	0.73 (0.31)	0.31–1.69	0.463
	High number of cognitive activities × high	0.51 (0.22)	0.22–1.20	0.123
Cognitive activities × LIBRA_adj_^4^	High number of cognitive activities × high	0.86 (0.30)	0.43–1.71	0.672
Social memberships × sex^1^	High number of social memberships × female	1.03 (0.26)	0.62–1.70	0.925
Social memberships × education^2^	High number of social memberships × medium	1.79 (0.57)	0.96–3.32	0.066
	High number of social memberships × high	1.68 (0.56)	0.87–3.23	0.116
Social memberships × wealth^3^	High number of social memberships × medium	1.09 (0.34)	0.59–2.00	0.793
	High number of social memberships × high	1.06 (0.35)	0.56–2.02	0.853
Social memberships × LIBRA_adj_^4^	High number of social memberships × high	0.84 (0.22)	0.51–1.39	0.494
Social participation × sex^1^	High frequency of social participation × female	1.18 (0.24)	0.86–1.63	0.296
Social participation × education^2^	High frequency of social participation × medium	1.74 (0.60)	0.88–3.42	0.111
	High frequency of social participation × high	1.64 (0.59)	0.81–3.35	0.171
Social participation × wealth^3^	High frequency of social participation × medium	1.80 (0.63)	0.91–3.56	0.093
	High frequency of social participation × high	1.45 (0.53)	0.71–2.98	0.307
Social participation × LIBRA_adj_^4^	High frequency of social participation × high	1.47 (0.41)	0.86–2.53	0.160

HR, hazard ratio; s.e., standard error; LIBRA_adj_, LIfestyle for BRAin health score without the weight for cognitive activity.

1 Adjusted for age, education, wealth and LIBRA_adj_ scores.

2 Adjusted for age, sex, wealth and LIBRA_adj_ scores.

3 Adjusted for age, sex, education and LIBRA_adj_ scores.

4 Adjusted for age, sex, education and wealth.

### Interactions

We first tested the interaction between cognitive activity and the social activity variables. Neither the interaction between cognitive activities and social memberships (*χ*^2^ = 0.19; df *=* 1; *p* = 0.661) nor the interaction between cognitive activities and social participation (*χ*^2^ = 0.07; df = 1; *p* *=* 0.798) were statistically significant. Next, the interactions between the three activity variables and the other risk factors were assessed (Table 3). We did not observe significant interactions between cognitive activities, social memberships or social participation with sex, education, wealth and LIBRA_adj_ scores.

### Sensitivity analyses

Different sensitivity analyses were conducted to test the robustness of the associations outlined above. First, cognitive activities, social memberships and social participation were included as continuous variables. In the separate models, each increase in the respective activity domain was significantly associated with lower dementia risk, independent of the covariates. However, in the joint model, cognitive activities were significantly associated with dementia, while social memberships and social participation were not.

Next, multiple imputation (White *et al*., 2011) was used to impute missing values of the activity variables, education, wealth and LIBRA_adj_ factors. Multiple imputation by chained equations (MICE) was chosen, as this method allows for missing data on multiple variables (van Buuren and Groothuis-Oudshoorn, 2000). Ten imputed datasets were created and combined using Rubin's rules (Rubin, 1996). The results of both the separate and joint models did not differ from the primary analyses.

In order to assess the possibility of reversed causality, we divided survival time into two parts, representing up to five years (incident dementia *n* = 147) and more than five years after baseline (incident dementia *n* = 213) and repeated the analyses with model 4. While in the first 5-year period, engagement in cognitive activities was strongly related to dementia risk in the separate model (HR = 0.42), it was not significant anymore in the second period (HR = 0.82). However, HRs for social memberships were comparable in both periods (HR first period = 0.69; HR second period = 0.70). For social participation, the association was stronger in the second period (HR = 0.77) as opposed to the first (HR = 0.85).

## Discussion

In this prospective cohort study, the association between a cognitively and socially active lifestyle and incident dementia was assessed. Considering the multifacetedness of activity engagement, this study aimed at further disentangling the unique contributions of three activity domains on dementia risk. When studied separately, people who reported higher engagement in cognitive and social activities showed a lower risk for developing dementia as compared to people who reported lower engagement. These associations remained significant when adjusting for non-modifiable (age, sex, education, wealth) and modifiable (LIBRA_adj_ scores) dementia risk factors. In addition, they did not interact with the latter, suggesting truly independent associations with dementia over and above other modifiable and non-modifiable risk factors. Furthermore, in the joint model including all three main predictors, associations for cognitive activities and social memberships and dementia risk were independent of each other. However, associations between social participation and dementia risk were attenuated and non-significant when considering the other activity domains.

These findings align with the growing body of literature underlining the relationship between engagement in cognitively stimulating and social leisure activities and dementia risk. This includes evidence of both cohort (Scarmeas *et al*., 2001; Wang *et al*., 2002; Fratiglioni *et al*., 2004; Karp *et al*., 2006; Foubert-Samier *et al*., 2014; Almeida-Meza *et al*., 2021) and case-control studies (e.g. Fritsch *et al*., 2005; Lindstrom *et al*., 2005).

Cognitive leisure activities as well as social engagement have been suggested as socio-behavioural proxies for cognitive reserve, a heuristic concept proposed for explaining interindividual differences in cognitive functioning in the light of similar neuropathological burden (Stern, 2002; Stern and Barulli, 2009; Stern *et al*., 2020). The underlying mechanisms behind cognitive reserve are largely unknown. However, changes in functional brain connectivity, especially in medial temporal and frontal regions, have been suggested (Anthony and Lin, 2018). Our findings fit with the idea of cognitive and social activities as being suitable proxies for cognitive reserve and show that they are independent of education and other dementia risk factors.

We also found that in the joint model, cognitive activities and social memberships were independently related to dementia risk, though the association between social memberships and dementia became modestly attenuated. Interestingly, this was also the case for cognitive activities, albeit to a lesser degree. Our findings align with those of Karp *et al*. (2006), who reported that mental and social components of activities were protective against dementia, independent of each other and after adjustment for covariates. On the other hand, social participation was not associated with dementia risk independent of cognitive activities and social memberships. The association between social participation and dementia was mainly attenuated by adding cognitive activities to the model and less so by adding social memberships. While the memberships of clubs and organisations and social activity engagement might be conceptually different entities, there may still be considerable overlap between the two. Our definition of social participation included activities such as going to museums and theatres, which often have both a social (going there with others) and cognitively stimulating component. Therefore, it does not come as a surprise that its association was explained by the other activities. Indeed, the three activity domains may lie on each other's causal pathway, in that memberships in more associations actually lead to more social participation, and they might partly relate to the same latent construct (cognitive reserve). Hence, the joint model might be over adjustment (Schisterman *et al*., 2009).

While the current study consistently found positive prospective correlations between cognitive and social activity engagement and dementia risk over 11 years and across the different models, the risk for reversed causality cannot be entirely excluded. A large-scale longitudinal study of 851 307 women in the UK (Floud *et al*., 2021) found that cognitive and social activities were associated with lower incident dementia within the first 4 years of follow-up, but the association diminished during the following 5–9 years of follow-up and was absent after more than 10 years of follow-up. The authors concluded that decreased activity engagement might actually be the result of impending dementia rather than a protective factor. Likewise, Eriksson Sörman *et al*. (2014) reported that associations between social activity engagement and dementia were only significant in the first 5 years after baseline and disappeared thereafter. In the current study, engagement in cognitively stimulating activities was only strongly related to dementia within the first but not the second 5-year period, giving rise to the possibility of reversed causality, which is in line with the findings of Floud *et al*. (2021). However, contrary to both Floud *et al*. (2021) and Eriksson Sörman *et al*. (2014), HRs between social memberships, social participation and dementia remained similar across both time periods, which may still support the possibility of a protective effect of social engagement for dementia. The differences in findings between the latter (Eriksson Sörman *et al*., 2014) and the current study could also have arisen through differences with regard to the operationalisation (e.g. frequency of engagement *v*. diversity of activities) and classification of activities as predominantly mental or social. Overall, future prospective research with sufficient follow-up should address reversed causality with regard to the activity engagement–dementia association, also in order to potentially exploit such directionality, for instance, for the purpose of an early dementia detection method.

Lastly, this study did not find significant interactions between cognitive activities, social memberships or social participation with other modifiable and non-modifiable dementia risk factors. Consequently, activity engagement might be equally beneficial for people of different ages, sex, socioeconomic strata or risk groups, which can be taken into account when designing and refining dementia prevention strategies. Contrary to our findings, a recent study using data from the Swedish National Study on Aging and Care – Kungsholmen found that engagement in an active lifestyle moderated the association between diabetes and dementia (Marseglia *et al*., 2020). However, the authors used a composite measure of education, work complexity, leisure activities and social network to assess an ‘active life’, which makes a direct comparison with the current study on individual components less straightforward.

### Strengths and limitations

This study has a number of strengths, including an extended period of follow-up in a large and representative sample of the British population and a high response rate, as well as a large number of modifiable dementia risk factors and plausible covariates included in the analyses. However, it also has some limitations. First, the sample was restricted to those without missing information on dementia incidence, LIBRA_adj_ factors and education (28% missing data). This might have led to the selection of a generally healthier sample and thus to an underestimation of the true association between activity engagement and dementia. The loss of follow-up of some participants due to the longitudinal nature of this study could have further amplified that. It is noteworthy that missing values of LIBRA_adj_ factors have been kept to a minimum by resorting to information of previous and subsequent waves. In addition, outcomes of the multiple imputation showed that results are stable compared to the primary analyses. Next, information on the activity variables was missing for some participants. Participants with missing information differed from the analytical sample on various key variables outlined above, which could have potentially induced bias. Moreover, the creation of composite scores for cognitive activity, social activity and social participation was done based on face validity and previous studies in ELSA and not on factor analysis. Due to the potential overlap, the individual contribution to explained dementia risk per activity domain might have been masked. In addition, dementia diagnoses, information on wealth, part of the LIBRA_adj_ factors and engagement in activities were based on self or caregiver reports. This may be a source for response bias and exposure misclassification. Lastly, there may be other factors not considered, which may drive or confound the associations observed in this study, such as occupational complexity, parental SES, parental education or parental lifestyle. Despite the limitations, this study adds to the growing body of research demonstrating the potentially beneficial effects of engagement in cognitive and social leisure activities on dementia risk.

## Conclusion

It was shown that cognitive and social activities were associated with dementia risk independent of various modifiable and non-modifiable risk factors. While activities partly overlap, both could equally play a role in programmes designed to decrease dementia risk. People with differing risk profiles may potentially benefit from a cognitively and socially stimulating lifestyle; therefore, targeted interventions should approach everybody equally. Future studies should preferably also further extend follow-up periods to minimise the risk for reversed causality.

## Data Availability

Data of the English Longitudinal Study of Ageing can be accessed freely via the UK data service (https://beta.ukdataservice.ac.uk/datacatalogue/series/series?id=200011). Upon reasonable request, data analysis protocols are available from the corresponding author.

## Supplementary material

For supplementary material accompanying this paper visit https://doi.org/10.1017/S204579602100069X. click here to view supplementary material
