# The role of cognitive and social leisure activities in dementia risk: assessing longitudinal associations of modifiable and non-modifiable risk factors – ERRATUM

**DOI:** 10.1017/S2045796022000038

**Published:** 2022-04-28

**Authors:** L. A. Duffner, K. Deckers, D. Cadar, A. Steptoe, M. de Vugt, S. Köhler

During final typesetting, a letter was inadvertently deleted changing the meaning of the title and this has since been rectified. Cambridge University Press apologise for this error.
