# Supplementary material for: The role of cognitive and social leisure activities in dementia risk: assessing longitudinal associations of modifiable and on-modifiable risk factors
Source: Epidemiol Psychiatr Sci. 2022 Jan 11;31:e5. doi: 10.1017/S204579602100069X (PMC8786616; doi:10.1017/S204579602100069X)
Supplement: Supplementary file 1 [file S204579602100069Xsup001.docx]

**Supplementary materials**

Supplementary Table 1: *Panel attrition of the analytical sample between Wave 4 and Wave 9.*

| **Wave** | 4 | 5 | 6 | 7 | 8 | 9 |
| --- | --- | --- | --- | --- | --- | --- |
| **N** | 7917 | 7720 | 7192 | 6450 | 5759 | 4977 |
| **% of wave 4 sample** | 100 | 97.5 | 90.8 | 81.4 | 72.7 | 62.8 |
|  | | | | | | |

Supplementary Table 2 – Operationalization of LIBRA factors and construction of the adjusted LIBRA score

| **LIBRA factor** | **Operationalization** | **Weight** |
| --- | --- | --- |
| Heart disease | Physician’s diagnosis of angina pectoris or myocardial infarction. | +1.0 |
| Diabetes (type-2)^*^ | Blood glycated hemoglobin level ≥ 48 mmol/mol (6.5%) according to the WHO guidelines [1]. | +1.3 |
| Hypercholesterolemia^*^ | Total cholesterol level of ≥5.0 mmol/L and low-density lipoprotein of ≥3.0 mmol/L, following the guidelines of the National Health Service UK [2]. | +1.4 |
| Hypertension^*^ | Mean systolic blood pressure ≥140 mm Hg or mean diastolic blood pressure ≥90 mm Hg [3]. | +1.6 |
| Depression^*^ | Total score on the 8-item Centre of Epidemiological Studies Depression (CES-D) scale of 3 or greater (range: 0-8) [4,5]. | +2.1 |
| Obesity | Established cut-offs according to the WHO guidelines [6]. Waist circumference (men: > 102 cm; women: > 88 cm) and waist-to-hip ratio (men: >90; women: >85) were only used if data on body mass index (BMI ≥ 30) was missing. | +1.6 |
| Smoking | Self-reported current smokers or non-smokers. | +1.5 |
| Low-to-moderate alcohol use | Self-reported frequency of any alcohol consumed in the past 12 months. Low-to-moderate alcohol use was defined as 1-14 glasses per week according to recent UK alcohol guidelines [7]. | -1.0 |
| Physical inactivity | Self-reported engagement in vigorous, moderate or mild physical activity during leisure time (more than once per week, once per week, one to three times per month, hardly ever). Participants were dichotomized into physically active (≥1/week) or physically inactive (1-3 times/month, hardly ever/never). | +1.1 |
| Healthy diet | Reported amount of fruits and vegetables consumed by the participant the previous day. A healthy diet was defined as consuming five or more portions of fruits and vegetables on a daily basis [8]. | -1.7 |

**REFERENCES**

[1] World Health Organization (2011) *Use of Glycated Haemoglobin (HbA1c) in the Diagnosis of Diabetes Mellitus: Abbreviated Report of a WHO Consultation.* World Health Organization, Geneva.

[2] National Health Service. High cholesterol. http://www.nhs.uk/Conditions/Cholesterol/Pages/Introduction.aspx. Accessed 27-09-2016.

[3] Guidelines Subcommittee (1999) 1999 World Health Organization-International Society of Hypertension Guidelines for the Management of Hypertension. *J Hypertens* **17**, 151-183.

[4] Radloff LS (1977) The CES-D Scale: A Self-Report Depression Scale for Research in the General Population. *Appl Psychol Measure* **1**, 385-401.

[5] Turvey CL, Wallace RB, Herzog R (1999) A revised CES-D measure of depressive symptoms and a DSM-based measure of major depressive episodes in the elderly. *Int Psychogeriatr* **11**, 139-148.

[6] World Health Organization (2008) *Waist circumference and waist–hip ratio: Report of a WHO expert consultation.* World Health Organization.

[7] Department of Health (2016) *Alcohol guidelines review — report from the guidelines development group to the UK Chief Medical Officers.* Department of Health.

[8] National Health Service (2009) *5 A Day.* NHS, London.
